# Measuring and exploring mental health determinants: a closer look at co-residents’ effect using a multilevel structural equations model

**DOI:** 10.1186/s12874-022-01711-9

**Published:** 2022-08-31

**Authors:** Hend Gabr, Mohammed Baragilly, Brian H. Willis

**Affiliations:** 1 Department of Mathematics, Insurance, and Statistics, Faculty of Commerce, Menoufia University, Shebeen El-Kom, Menoufia, Egypt; 2 Department of Mathematics, Insurance, and Applied Statistics, Helwan University, Helwan, Egypt; 3 Istitute of Immunology and Immunotherapy, University of Birmingham, Edgbaston, Birmingham, B15 2TT UK; 4 Institute of Applied Health Research, University of Birmingham, Edgbaston, Birmingham, B15 2TT UK

**Keywords:** Multilevel structural equations, Psychiatric morbidity, GHQ-12

## Abstract

**Objective:**

Previous research has demonstrated that individual risk of mental illness is associated with individual, co-resident, and household risk factors. However, modelling the overall effect of these risk factors presents several methodological challenges. In this study we apply a multilevel structural equation model (MSEM) to address some of these challenges and the impact of the different determinants when measuring mental health risk.

**Study design and setting:**

Two thousand, one hundred forty-three individuals aged 16 and over from 888 households were analysed based on the Household Survey for England-2014 dataset. We applied MSEM to simultaneously measure and identify psychiatric morbidity determinants while accounting for the dependency among individuals within the same household and the measurement errors.

**Results:**

Younger age, female gender, non-working status, headship of the household, having no close relationship with other people, having history of mental illness and obesity were all significant (*p* < 0.01) individual risk factors for psychiatric morbidity. A previous history of mental illness in the co-residents, living in a deprived household, and a lack of closeness in relationships among residents were also significant predictors. Model fit indices showed a very good model specification *(CFI* = *0.987, TLI* = *0.980, RMSEA* = *0.023, GFI* = *0.992)*.

**Conclusion:**

Measuring and addressing mental health determinants should consider not only an individual’s characteristics but also the co-residents and the households in which they live.

**Supplementary Information:**

The online version contains supplementary material available at 10.1186/s12874-022-01711-9.

## Introduction

Mental health is a wide-ranging issue that affects a significant segment of populations around the world [1]. In 2017, 792 million people worldwide were estimated as living with mental disorder (roughly 10.7% of the global population) [2]. Untreated mental disorders accounted for 13% of the total global burden of disease in 2011 and it is projected that by 2030 depression will be the leading cause of disease burden globally [1]. In the Health Survey for England (HSE) 2014, 26% of respondents reported having been diagnosed with at least one mental disorder in their lifetimes, while a further 18% of adults reported having experienced undiagnosed psychiatric morbidity [3].

There are a number of risk factors associated with mental illness in an individual. Several studies have found that mental health disorders are more prevalent among younger age groups [4, 5]. Many factors that have been shown to be associated with mental health symptoms such as unemployment, economic hardship [5] and the burden placed upon younger heads of the household to support their older relatives [6] are more prevalent among younger than older subjects. Taking the responsibility role in the family makes the head of the household more vulnerable to mental illness [7]. Not working is also a risk factor for mental illness [5, 8, 9] even though other studies have pointed out the stressful impact of work on mental illness [10, 11]. Another crucial risk factor of mental illness is having a history of a common mental disorder [12]. A recent study showed that more than half of mental health patients in the UK experience relapses [13].

The mental health of co-residents is also of interest to researchers. Studies have shown that mental illness, such as depression, can have a negative effect on family relationships [14]. Other studies have shown that living with individuals with mental illness may impose financial stress on the family [15]. In contrast, individuals who have close relationships with other people are less likely to have mental health disorders [16]. Social relations are in many ways important for mental health as a protecting factor in psychosocial crisis situations and strain [5, 17, 18].

Household characteristics, such as structural housing problems and being part of a low-income household have also been associated with poor mental health [4]. Using ‘an index of multiple deprivation’ to reflect different aspects of housing deprivation, it has been shown that the prevalence of mental illness is highest in the most deprived areas [19]. Living in a deprived area exposes people to a high number of stressors, such as, unsafe neighbourhoods and comparison of the self to others, which in turn, lead to stress and poor mental health [20, 21].

When investigating the mental health of populations, the population survey is a common tool used for collecting data. Surveys such as the HSE provide rich datasets on the everyday circumstances of a random sample of households in England and in 2014 it focussed on mental health. However, using data from surveys to develop statistical models can raise a number of issues. For example, how we measure mental health and psychiatric morbidity particularly in large scale surveys requires careful consideration since the resources may not be available for expert interviews by clinical psychologists or psychiatrists. Overcoming the lack of a ‘gold standard’ in measuring mental health presents a methodological challenge.

Furthermore, as the evidence above highlights, there are potentially a multitude of sources and determinants of an individual’s mental health that any modelling approaches to mental health data should, in principle, incorporate. For instance, it may be expected that the responses to survey questions from those in the same household are more likely to be similar, since they are more likely to be subject to common influences that are not shared among individuals of different households [22]. Yet these interactions between individuals in the same household and household-level factors have yet to be adequately dealt with in statistical modelling approaches to mental health survey data proposed thus far.

In order to explore some of these methodological issues requires a dataset that provides data on individuals, co-residents, and household characteristics. To this end we will use the HSE 2014 dataset and apply a multilevel structural equations model (MSEM) for modelling mental health in the community.

## Methods

### Dataset

The Health Survey for England (HSE) is an annual survey that provides information about adults’ and children’s health in England. In 2014, the topic of focus was mental health [3] and as it is yet to be repeated, this remains the most recent HSE that focussed on mental health. Questions covering experience of mental health problems and the 12-item General Health Questionnaire (GHQ-12) questions [23] were directed to a total of 5,491 adults. There were no data on the mental of children in the household so in order to provide accurate results about the impact of co-residents’ mental health we considered only households in which all the members were adults. Moreover, single households were excluded as they have no co-residents. A total of 2143 individuals aged 16 and over representing 888 households with 2 or more adults were analysed.

### Outcome – measuring latent psychiatric morbidity

The GHQ-12 developed by Goldberg in the 1970s [23] is a widely used measure of psychiatric morbidity symptoms across populations. Although some researchers have asserted the multidimensionality of the GHQ-12 [4, 24–26], the responses to the questions are usually combined to construct a unidimensional score for the individual’s mental health based on a specific scoring method such as the bi-modal scoring style, the Likert scoring style and the C-GHQ method [27–29].

The present study considers three methodological issues with the GHQ-12 measure that require more investigation. The first is the treatment of measurement errors associated with each question, which may result from the ambiguous wording of the responses to the negatively phrased items [27, 30] and the carelessness of respondents in reading the questions [24]. These may result in a biased estimate of the outcome of interest and affect the utility of this measure [31].

Often the GHQ-12 is applied as a unidimensional measure in which the individual indicator scores are summed to give an overall score. Implicitly this assumes that each indicator of the GHQ-12 measure plays the same role in contributing to an individual’s psychiatric morbidity score by applying equal weighting to each indicator. Using a MSEM approach we may use the data to test this assumption and determine an appropriate weighting scheme.

The final issue is the dependency between individuals within the same household. As already described, individuals nested within the same household are more likely to be subject to common influences and ignoring this clustering structure may result in biased results and unrealistic standard errors for the estimated parameters [6, 32, 33]. These methodological issues can have important implications for inferences that are made and the conclusions that are drawn from a particular study.

Here we assume that the true psychiatric morbidity for an individual is a unidimensional score that is unknown and will be treated as a latent variable. We measure an individual’s latent psychiatric morbidity (the outcome) using the observed twelve items of GHQ-12 measure (Y1 to Y12). The responses to these questions are coded on an ordinal scale of 4 Likert-type responses. Table 1 gives the frequency of the outcome responses to the GHQ-12 in the HSE 2014 dataset. As expected, response 2 (“Same as usual”, “About same as usual” or “No more than usual”) is the most frequent response for 10 of the 12 questions. The question which returned the highest for response 4 (“Much less than usual” or “Much more than usual”) was “Able to enjoy day-to-day activities” (2.8%). In addition, response 3 (Less so than as usual” or “Rather more than usual”) also indicates the presence of psychiatric morbidity and was highest for “Felt constantly under strain” (15.8%), “Lost sleep over worry” (12.8%) and “Able to enjoy day-to-day activities” (12.4%).

**Table 1:** Outcomes: GHQ-12 questionnaire responses and characteristics

		**Responses**				
		**1**	**2**	**3**	**4**	**Total**
**Variable**		**N (%)**	**N (%)**	**N (%)**	**N (%)**	**N (%)**
Y_1_	Ability to concentrate	52 (2.6)	1748 (87.4)	181 (9.1)	18 (0.9)	1999 (100)
Y_2_	Felt playing useful part in things	191 (9.6)	1596(80.1)	164 (8.2)	42 (2.1)	1993(100)
Y_3_	Felt capable of making decisions	133(6.7)	1747 (87.4)	108 (5.4)	12 (0.6)	2000 (100)
Y_4_	Able to enjoy day-to-day activities	95 (4.8)	1598 (80.1)	247(12.4)	55 (2.8)	1995 (100)
Y_5_	Been able to face problems	84 (4.3)	1753 (89.2)	109 (5.5)	19 (1.0)	1965 (100)
Y_6_	Been feeling reasonably happy	189 (9.6)	1612 (81.9)	137(7.0)	31 (1.6)	1969 (100)
Y_7_	Lost sleep over worry	673 (33.7)	1025 (51.2)	255 (12.8)	47 (2.4)	2000 (100)
Y_8_	Felt constantly under strain	541 (27.1)	1096 (54.9)	315 (15.8)	44 (2.2)	1996 (100)
Y_9_	Felt could not overcome difficulties	760 (38.1)	1059 (53.1)	140 (7.0)	34 (1.7)	1993 (100)
Y_10_	Been feeling unhappy and depressed	811 (41.2)	891 (45.2)	228 (11.6)	40 (2.0)	1970 (100)
Y_11_	Been losing confidence in self	886(45.0)	873 (44.4)	174 (8.8)	34 (1.7)	1967 (100)
Y_12_	Been thinking of self as worthless	1349 (68.4)	502 (25.5)	91 (4.6)	29 (1.5)	1971 (100)

*Key to responses*

Response 1 “Better than usual” (Y_1_); “More so than usual” (Y_2_-Y_6_); “Not at all” (Y_7_-Y_12_)

Response 2 “Same as usual” (Y_1_-Y_5_); “About same as usual” (Y_6_); “No more than usual” (Y_7_-Y_12_)

Response 3 “Less so than usual” (Y_1_-Y_6_); “Rather more than usual” (Y_7_-Y_12_)

Response 4 “Much less than usual” (Y_1_-Y_6_); “Much more than usual” (Y_7_-Y_12_)

### Predictors

We include the co-residents’ experience of common mental disorders throughout their life as the main explanatory variable. It is measured as a dichotomous variable where the value 1 means the individual is living with at least one co-resident who has previously experienced at least one common mental disorder and zero means none of the co-residents has ever experienced a common mental disorder.

Other individual-level predictors include age, gender, work status, being the head of the household, obesity, individual’s history of common mental disorders and having a close relationship with other people. At the household-level we control for household deprivation and the household’s close relationship with other people. Table 2 defines the predictors at the individual level and at the household level.

**Table 2:** Predictor variables at the individual and the household level – definitions

**Predictors measured at the individual level**	
X^(co)^: Co-resident’s experience of common mental disorder	A binary variable where 1 means a presence of at least one co-resident who has ever experienced any common mental disorders and zero means none of the co-residents has ever experienced common mental disorders
X_1_: Age	A continuous variable represents individual’s age in years
X_2:_ Male	A binary variable where 1 indicates male and 0 indicates Females
X_3:_ Working status	A binary variable where 1 indicates the person is working and 0 indicates non-working
X_4:_ individual’s history of common mental disorder	A binary variable where 1 indicates the person has experienced a common mental disorder during his/her life and 0 indicates otherwise
X_5:_ Headship of the household	A binary variable where 1 indicates the person is a householder and 0 otherwise
X_6:_ having close relationship with other people	An ordinal variable ranges from 1 to 5 where 5 represents closer relationship with other people
X_7:_ Obesity	A continuous variable represents individual’s body mass index
**Predictors measured at the household-level**	
Z_1_: Household Deprivation index	An ordinal variable represents quintiles of deprivation, ranked in ascending order of deprivation score where quintile 1 means least deprived
Z_2_: Household’s close relationship with other people	A continuous variable represents a score ranges from 1 to 5 where 5 represents closer relationship with other people

### Statistical analysis

Multilevel structural equations modelling is a general framework that allows the estimating of latent variables in clustered data such as for individuals within and between households [34].

**Fig. 1:**
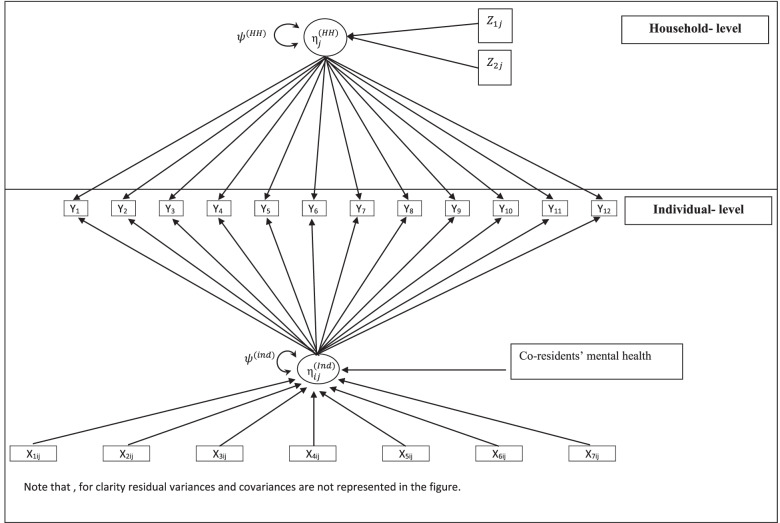
The Framework and the graphical notation of the model

Figure 1 gives a graphical representation of the model. We followed graphical conventions originally recommended in [34] which bears a one-to-one correspondence with the underlying statistical model. The two blocks labelled ‘individual-level’ and ‘household-level’ represent the two levels of data and within each block, rectangles signify the observed variables whilst circles signify the latent factors.

The two sets of observed variables are:

- (a) *Dependent variables*—the GHQ-12 indicators $({Y}_{pij})$, where *p* = *1, 2…,12* used to measure the latent psychiatric morbidity for individual *i* in household *j*.
- (b) *Predictors*${\mathrm{X}}_{ij}^{(co)}$, ${X}_{kij}$ and ${Z}_{rj}$ are used to explain the variability in an individual’s latent psychiatric morbidity at both levels (i.e. within and between households) respectively, where *k* = 1,2,…,7; and *r* = 1,2.

The latent factors, ${\upeta }_{ij}^{(Ind)}$ and ${\upeta }_{j}^{(HH)}$, represent the latent psychiatric morbidity for the individual and household, respectively. A line with a single arrowhead denotes the effect of one variable on another and a curved double-ended arrow represents the variance–covariance structure of the error terms. $\psi$ refers to the residual variance of the latent factors, and $\theta$ refers to the residual variance of the observed variables (not shown).

At the individual-level, there are twelve indicators ( ${Y}_{pij}$) that load onto a single factor ${\upeta }_{ij}^{(Ind)}$ (individual psychiatric morbidity score) and as such this treats the GHQ-12 as a unidimensional measure in line with confirmatory factor analysis [27–29]. Further, we have co-residents’ history of mental disorders ${\mathrm{X}}_{ij}^{(co)}$ which is our main explanatory variable of interest in addition to seven predictors at the individual-level and two predictors at the household-level described in Table 2.

The analyses were carried out using the Lavaan package (version 0.6–8) which can deal with categorical variables [35] in the R environment [36]. The parameters are estimated using diagonally weighted least squares method. To assess the fit of the entire model, there is no single most powerful fit index in SEM as there are for other techniques, so we used (a) the goodness-of-fi t index (GFI; scaled from 0 to 1, with acceptable fit having values larger than 0.95), (b) the root mean square error of approximation (RMSEA; acceptable fit with values less than 0.08 or 0.05), and (c) the comparative fit index (CFI; scaled from 0 to 1, with acceptable fit having values larger than 0.90 or 0.95) [34]. Moreover, the Akaike information criterion (AIC) and the Bayesian information criterion (BIC) are used to compare different possible models and determine which one is the best fit for the data. Missing values are handled using listwise deletion. For model modification, alternative models were generated and then fitted to the same data matrix and modification indices are calculated. These indices estimate the amount by which the overall model chi-square would decrease if a previously-fixed-to-zero parameter were freely estimated [34]. In the next section, the results of the modelling are presented. The model is given in more detail in an online appendix.

### Hypotheses tests

Based on the model described in Fig. 1, we proposed the following hypotheses tests. For the measurement component of the model, we test the relevance of the 12 observed variables in explaining the latent factor. So, we test H_p_: ${\lambda }_{p}$>0, for *p* = 1,2….12. We then examine whether the variance of household-level residuals differs significantly among households to verify the need for multilevel modelling. More specifically, for the household’s psychiatric morbidity latent factor, we test H_13_: var (${\upeta }_{j}^{\left(HH\right)}$) > 0 against the null hypothesis of no variation. We expected to find evidence of var (${\upeta }_{j}^{\left(HH\right)}$) > 0, which would emphasise the importance of accounting for the household effect.

We also test the effect of a set of demographic and socio-economic variables on psychiatric morbidity at the individual level, such as H_14_: age < 0; H_15_: male gender < 0; H_16_: working < 0; H_17_: being householder > 0; H_18_: having history of mental illness > 0; H_19_: having close relationship with other people < 0; and H_20_: obesity > 0 against the null hypothesis of no effect. Meanwhile, among the factors that would influence psychiatric morbidity, we are particularly interested in testing the effect of living with at least one co-resident who has previously experienced mental illness. Thus, against the null hypothesis of no effect, we test H_21_: Co-residents’ history of common mental disorder > 0. We expected to reject the null hypothesis of no effect and find evidence to support the alternative hypotheses H_21_.

Finally, as far as the effect of household-level variables are concerned, we expected to find that there is a significant relationship of living in a deprived area in increasing individual psychiatric morbidity and household’s closeness to other people in decreasing the outcome. Thus, we test the hypothesises H_22:_ deprivation > 0 and H_23_: closeness to other people < 0 against the null hypothesis of no effect.

## Results

### Data characteristics

In Table 3 a breakdown of the sample data on the predictors is also given. What is clear is that most of the interviews appear to have been conducted with the ‘head of household’ (82.8%). The average age of subjects was 56 and around a quarter of subjects had experienced a common mental health disorder (X_4_). A 27.6% of the sample lived with at least one individual who had a previous history of mental illness (X^(co)^). Only 13% of the dataset was collected from the most deprived areas.

**Table 3:** Predictor variables—characteristics

**Predictors measured at the individual level**	
* Binary variables*	Sample total; % with value 1
X^(co)^: Co-resident’s experience of common mental disorder	*N* = 2143; 27.6%
X_2_: Male	*N* = 2143; 49.7%
X_3_: Working status	*N* = 2143; 46.4%
X_4_: individual’s history of common mental disorder	*N* = 2143; 24.1%
X_5_: Headship of the household	*N* = 2143; 82.8%
* Ordinal variables*	Sample total; frequencies
X_6_: having close relationship with other people	*N* = 1996; none = 22, rarely = 102, some of the time = 616, often = 886, All the time = 370
* Continuous variables*	Sample total; mean (SD)
X_1_: Age	*N* = 2143, 55.76 (18.38)
X_7_: Obesity	*N* = 2042, 26.72 (5.3)
**Predictors measured at the household-level**	
* Ordinal variables*	Sample total; frequencies
Z_1_: Household Deprivation index	*N* = 2143; Quintiles Q1 = 549, Q2 = 500, Q3 = 458, Q4 = 352, Q5 = 284
* Continuous variables*	Sample total; mean (SD)
Z_2_: Household’s close relationship with other people	*N* = 1933; 3.749 (0.632)

*Key*: *SD* Standard deviation

### Intra-class correlation of the psychiatric morbidity factor and the observed indicators

To ascertain what proportion of the variability in the latent psychiatric morbidity may be assigned to the household, we first estimated the null (structural base line) model to estimate the proportion of variability in each of the twelve observed indicators due to clustering. This null model is a model with all the measurement parameters estimated, but the structural parts (relationships between variables) are constrained to zero. The intra-class correlations (ICCs) ranged from 0.042 to 0.219 indicating that household differences explained between 4.2 and 21.9% of the variability in these outcomes. Moreover, the ICC for the latent psychiatric morbidity showed that 23.5% of variability in individual psychiatric morbidity is due to differences between households. In practical terms, the ICC represents the variability that is potentially explainable by household predictors while the complements, which range between 78.1 and 95.8%, are the proportions of variability due to individual differences within households.

### Results of the measurement component

We start with measuring the individuals’ and households’ latent psychiatric morbidity. For each of these parameters, the modification index was computed in order to provide suggestions for a better fit of the specified model [37]. Alternative models were generated and then fitted to the same data matrix and modification indices were calculated. These indices estimate the amount by which the overall model chi-square would decrease if a previously-fixed-to-zero parameter were freely estimated. We allowed the error covariances between each pair of the psychiatric morbidity indicators to be freely estimated in a stepwise manner. When all psychiatric morbidity indicators error covariances were not constrained to zero, the model gave the best results in terms of the values of fit indices (CFI, TLI, RMSEA, GFI) and model performance measures (AIC and BIC).

Thus, the Modification Indices suggested a potential model re-specification by allowing for the existence of an error covariance between the twelve indicators of psychiatric morbidity. The existence of this covariation is justified and may be due to a common method variance (the tendency to respond in the same way to similarly worded items [27]). Additionally, the factor loadings at the household level were assumed to be invariant meaning that the effect of a household latent factor on a given individual outcome was not different from the effect of individual psychiatric morbidity factor on the same outcome. Thus, the level-2 latent factor is thought of as a random intercept for the level-1 latent variable [34].

The factor loading estimates for the twelve indicators are given in Table 4. The first factor loading is fixed to 1.0 to link the scale of the unobserved psychiatric morbidity factor to that of the first observed variable. This provides the weighting scheme for the GHQ-12 when measuring psychiatric morbidity. All the factor loadings were highly significant (*p* < 0.01). To evaluate the model fit we applied CFI, TLI, RMSEA and GFI [38]. This revealed a satisfactory level of overall fit with CFI = 0.987, TLI = 0.980, RMSEA = 0.023 and GFI = 0.992. In contrast, the corresponding figures for the structural baseline model (no predictors nor assumed covariances between the GHQ-12 indicators) were CFI = 0.843, TLI = 0.826, RMSEA = 0.081and GFI = 0.634, respectively.

**Table 4:** Factor loading (weighting) of observed indicators of individual’s and households’ psychiatric morbidity

${Y}_{pij}^{(ind)}$	${\lambda }_{p}^{(ind)}, {\lambda }_{p}^{(HH)}$	95% Confidence interval		Standardized ${\lambda }_{p}^{(ind)}, {\lambda }_{p}^{(HH)}$
		Lower	Upper	
**Ability to concentrate**	1	1	1	0.134
**Felt playing useful part in things**	1.415	1.049	1.781	0.189
**Felt capable of making decisions**	0.954	0.698	1.210	0.128
**Able to enjoy day-to-day activities**	1.837	1.437	2.237	0.246
**Been able to face problems**	0.964	0.707	1.221	0.129
**Been feeling reasonably happy**	2.059	1.581	2.536	0.276
**Lost sleep over worry**	3.366	2.586	4.146	0.450
**Felt constantly under strain**	3.575	2.758	4.392	0.478
**Felt could not overcome difficulties**	3.482	2.701	4.263	0.466
**Been feeling unhappy and depressed**	4.516	3.516	5.517	0.604
**Been losing confidence in self**	4.225	3.276	5.173	0.565
**Been thinking of self as worthless**	3.634	2.797	4.47	0.486

All *p*-value = 0.000

### Results of the regression

**Fig. 2:**
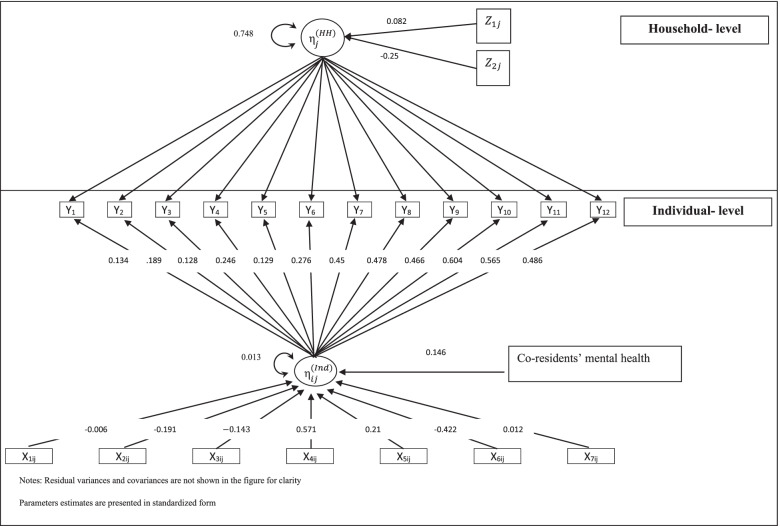
Graphical presentation of the results

The results in Table 5 and Fig. 2 show that all of the predictors are statistically significant. At the individual-level, if the effect of other predictors is held constant, then the results show that living with at least one co-resident with a history of a common mental disorder is associated with increasing the corresponding individual’s psychiatric morbidity score. In other words, living with others who suffer from psychiatric morbidity at a certain point in their lives is associated with an individual suffering from psychiatric morbidity symptoms. This result agrees with the findings of the other studies that show a significant association between partners’ mental health [39].

**Table 5:** Parameter estimates of psychiatric morbidity correlates (Eq. (4) of the Appendix)

**Individual-level variables**	${\varvec{\beta}}$	95% Confidence interval		Standardized ${\varvec{\beta}}$	Multicollinearity statistics	
		Lower	Upper		Tolerance	VIF
Co-residents’ history of common mental disorder	0.020^a^	0.005	0.034	0.146	0.924	1.082
Age	-0.001^a^	-0.001	0	-0.006	0.589	1.698
Male	-0.026^a^	-0.039	-0.013	-0.191	0.919	1.089
Householder	0.028^a^	0.009	0.047	0.210	0.789	1.268
Individual’s history of common mental disorder	0.076^a^	0.054	0.098	0.571	0.919	1.088
Working	-0.019^b^	-0.034	-0.004	-0.143	0.738	1.356
Closeness to other people	-.056^a^	-0.071	-0.042	-0.422	0.435	2.301
Obesity	0.002^a^	0	0.003	0.012	0.965	1.037
${\psi }^{(Ind)}$	0.013^a^	0.008	0.019	0.748		
${\psi }_{Null}^{(Ind)}$	**0.026**^a^					
**Household-level variables**	${\varvec{\gamma}}$					
Deprivation	0.004^c^	-0.001	0.009	0.082	0.976	1.025
Closeness to other people	-0.013^c^	-0.027	0.001	-0.250	0.972	1.028
${\psi }^{(HH)}$	0.003^a^	0.001	0.004	0.959		
${\psi }_{Null}^{(HH)}$	0.008^a^					

^a^ Significant at level *p* < 0.01

^b^ Significant at level *p* < 0.05

^c^ Significant at level *p* < 0.1

At the household-level, which implicitly allows investigating the effect of current co-residents’ mental health, the predictors of interest are statistically significant. Thus, being in a high quintile for the deprivation index (i.e., most deprived) was associated with a significant increase in an individual’s psychiatric morbidity score. Furthermore, the household’s close relationship with other people was associated with a significant decrease in an individual’s psychiatric morbidity.

Furthermore, we accounted for the effect of age, gender, headship of the household, history of mental illness, working status, having close relationship to other people and obesity. Results showed that increasing in age, being male, working and having a close relationship with other people decreases an individual’s psychiatric morbidity score. Other predictors that were found to increase psychiatric morbidity were: headship of the household, having a history of a common mental disorder, and obesity.

Two approaches were employed to examine multicollinearity among the predictors. First, pairwise correlations of the predictors were calculated. Statistically, a correlation coefficient of 0.90 and above indicates the presence of multicollinearity [40]. The following correlation matrix among the predictors showed that most of the correlations coefficients are very low. The highest correlation coefficient is 0.74 which is between having close relationship with others at the individual level and the household level.

$$\begin{bmatrix}1&&&&&&&&&\\0.12&1&&&&&&&&\\0.02&0.03&1&&&&&&&\\-0.06&-0.13&-0.48&1&&&&&&\\-0.19&0.14&0.02&0.02&1&&&&&\\0&-0.08&-0.07&0.43&-0.02&1&&&&\\0.05&0.04&0.04&0.1&0.03&0.08&1&&&\\-0.12&-0.03&-0.01&0.07&-0.07&0.04&-0.06&1&&\\0.08&0.06&0.02&-0.1&-0.01&-0.06&0.05&-0.04&1&\\-0.1&-0.1&-0.01&0.07&0&0.05&-0.07&0.74&-0.07&1\end{bmatrix}$$

Secondly, multicollinearity statistics were performed. Tolerance was examined for each predictor to examine the percent of variance in the predictor that cannot be accounted for by the other predictors while Variance Inflation Factor (VIF) measures how much the variance of the estimated regression coefficient is “inflated” by the existence of correlation among the predictor variables in the model. A VIF of 1 means that there is no correlation among the predictor and the remaining predictors, and hence the variance of this predictor is not inflated at all. In general, multicollinearity is considered a concern if the VIF is higher than 5 and the tolerance value is < 0.20 [40]. Table 5 indicates that multicollinearity is unlikely to be a concern among the predictors since all the VIF values were < 5 and tolerance values exceeded 0.20.

### The result of the random effect

For the remaining variance in the model, we calculated the percentage of explained variance at each level. For the predictors at the individual level $({\psi }_{Null}^{\left(Ind\right)}-{\psi }^{\left(Ind\right)})/{\psi }_{Null}^{(Ind)}$ equals 0.5. This demonstrated that the predictor variables added to the model at the individual-level explained 50% of the variance in psychiatric morbidity scores while the predictor variables at the household-level explained 62.5% of the variance ($({\psi }_{Null}^{\left(HH\right)}-{\psi }^{\left(HH\right)})/{\psi }_{Null}^{(HH)}$). Thus, the model explained a substantial proportion of the between-household variance.

## Discussion and concluding remarks

The question of whether household factors have a significant impact on an individual’s psychiatric morbidity, in part has implications which are both medical and societal. In this study we found that the mental health of individuals in the same household is significantly dependent on each other. This in part may be environmental due to the stresses brought from sharing the same environment and life events. It may also be due to genetic factors when individuals are related.

This source of variability raises several questions about the effect of co-residents on an individual’s mental health and poses a challenge on identifying reasons behind this variability. The contribution here is threefold. First, we account for two sources of variability, namely, the dependency between survey respondents and measurement errors in the GHQ-12 measure.

Secondly, in contrast to other studies that assign equal weights to each item of the GHQ-12 measure, we measured psychiatric morbidity using a statistical technique that allows the weights to vary according to the importance of each indicator in explaining the latent psychiatric morbidity.

Thirdly, we gave particular consideration to the effect that co-residents have on an individual’s psychiatric morbidity. For each individual we examined the effect of living with at least one co-resident who has previously experienced a mental illness on the outcome of interest. Furthermore, we identified two important predictors measured at the household-level that had a substantial effect on an individual’s psychiatric morbidity. These represented the household index of multiple deprivation and having a close relationship with other people.

Several studies found that living in a deprived area exposes people to a high number of stressors, such as, lack of personal safety, noise pollution, low social ranking, low self-esteem, and comparison of the self to others, which in turn, lead to stress and poor mental health [20, 21]. Furthermore, the household’s close relationship with other people was associated with a significant decrease in an individual’s psychiatric morbidity, which agrees with previous studies that shed the light on social capital as a protecting factor in psychosocial crisis situations and strain [17, 18].

We also identified a number of individual predictors that were shown to be associated with mental health and the results are broadly consistent with previous research. For example, being of a young age increases the risk of mental illness since many of the factors that have been shown to be associated with mental health symptoms such as unemployment, economic hardship [4, 5] and the burden placed upon younger heads of the household to support their older relatives [6] are more prevalent among younger than older subjects. However, the effect of age in our study is small compared with other predictors.

Our study is also in line with previous research in showing that being male is associated with a lower risk of mental illness [4, 9–11, 16, 41]. This observation is consistent with other research that has demonstrated women suffering higher levels of the stress hormone noradrenalin than men in the workplace [11], which is compounded by performing greater levels of unpaid work particularly in relation to the home and childcare [10, 11, 41].

Householders are often perceived by their families to be the main provider and such a role is associated with a greater risk of mental illness [7]. Our findings are consistent with this observation. The role of work in mental illness is complex. On the one hand, lack of work or not working has been shown to increase the likelihood of mental illness [5, 8, 9]; on the other hand, the work itself may be a source of stress that makes mental illness more likely [10, 11]. Our study demonstrated that not working was associated with a greater risk of mental illness.

Having a history of a common mental disorder had the largest effect on developing psychiatric morbidity in this study. This agrees with several studies that asserts the risk of relapse of mental illness [12, 13]. Obesity is associated with a heavy psychological burden including low mood, low self-esteem, poor quality of life, and body image dissatisfaction [42]. Our findings agree with previous research that obesity exerts a small but significant effect on mental health.

### Strengths and weaknesses

The strength of this paper is that we have demonstrated using multilevel structural equations model the determinants of psychiatric morbidity in individuals in the community. This would not have been possible without a rich dataset and the Health Survey for England 2014, which focusses on mental health, provides data on the GHQ-12, individual covariates, and co-residents in households. Thus, we were able to examine the nested structure of the data, the latent nature of psychiatric morbidity, the response bias, and the different weighting of each indicator in measuring the outcome.

The study also highlights the methodological issues when applying clinical psychometrics to individuals in order to measure their mental health. The use of the GHQ-12 to assess psychiatric morbidity is not without challenges particularly when determining a weighting scheme for the different indicators in addition to the well-known problem of common method variance.

However, there are also limitations regarding the data availability. Data on the experience of mental health problems were only available for adults aged 16 and over in the HSE dataset. Consequently, we could not neither examine the effect of having a child with mental illness on an individual’s mental health nor the effect of living with parents with a mental disorder on the child’s well-being. Such an analysis would provide important insights into the family dynamic on an individual’s mental health and is worthy of further research.

Another limitation is that we were unable to ascertain how long the household has been established or the movement of individuals between households from the data analysed. These too may affect the psychiatric morbidity measured in individuals. For example, a newly established household is unlikely to show the effects of its members on an individual’s psychiatric morbidity whilst the effects of a previous household on an individual’s psychiatric morbidity may hold for a long time. Nonetheless, the effect seems worthy of further investigation and potentially may inform how clinicians manage patients with mental illness.

The findings have implications for the design of surveys and interventions aimed at ascertaining and improving mental health at the household level. What was noticeable is the absence in the data of factors known to have an effect on an individual’s mental health such as death or serious illness of a family member, or a history of domestic violence or abuse.

Further research is needed to elicit the underlying drivers of the co-residents’ effect. Thus, the qualitative study of the co-residents’ mental health effect and how they vary over time may provide a clearer picture on causation. The inclusion of within-family dynamics effect in future models may also throw some light on this issue. The use of longitudinal data to monitor the change of individuals’ psychiatric morbidity requires further research and this will have implications for future models since the nesting structure will be more complicated as a result.

In summary, using a multilevel structural equation model on data acquired from a detailed household survey, we demonstrated the potential effect that co-residents may have on a person’s mental health in addition to a number of well recognised individual risk factors. To the best of our knowledge this is the first time such a model has been applied to this problem. 

## Supplementary Information

**Additional file 1.** Appendix - Model specification.

## Data Availability

All data used in the manuscript are available in the public domain at https://beta.ukdataservice.ac.uk/datacatalogue/studies/study?id=7919
