# Supplementary material for: Measuring and exploring mental health determinants: a closer look at co-residents’ effect using a multilevel structural equations model
Source: BMC Med Res Methodol. 2022 Aug 31;22:236. doi: 10.1186/s12874-022-01711-9 (PMC9429464; doi:10.1186/s12874-022-01711-9)
Supplement: Supplementary file 1 — Additional file 1. Appendix - Model specification. [file 12874_2022_1711_MOESM1_ESM.docx]

**Appendix - Model specification**

The basic structure of our model is that we have 12 psychiatric morbidity indicators and have 2143 individuals nested within 888 households. The model consists of a measurement component, which is set to measure individual’s psychiatric morbidity, and a structural component which considers the relationship between variables.

*A1.1 Measurement component*

First, we consider the psychiatric morbidity of individuals who are nested within the same household using the twelve indicators ($Y_{1}, Y_{2}{,\ldots Y}_{12}$) defined in table 1. We assume all twelve dependent variables represent a single unobserved or latent factor that determines an individual’s psychiatric morbidity status. The first set of structural equations is formulated to measure latent psychiatric morbidity at each level as follows:

$$Y_{pij}^{\left( Ind \right)}=\alpha_{p}^{\left( Ind \right)}+\lambda_{p}^{\left( Ind \right)}\eta_{ij}^{\left( Ind \right)}+\lambda_{p}^{\left( HH \right)}\eta_{j}^{\left( HH \right)}+e_{pij}^{\left( Ind \right)} (1)$$

$$\forall i=1,2,..,n ; j=1,2,\ldots,N;p=1,2,\ldots,12$$

Where, $Y_{pij}^{(Ind)}$ is the observed variable $p$ for the *i*^th^ individual of household *j*, $\alpha_{p}^{(Ind)}$ is the intercept term for variable *p*, $\lambda_{p}^{(Ind)}$, $\lambda_{p}^{(HH)}$ are the factor loadings of the observed variable $p$ on the latent individual psychiatric morbidity ($\eta_{ij}^{(Ind)}$), and latent household psychiatric morbidity ( $\eta_{j}^{(HH)}$) respectively that are assumed to be fixed across levels, $e_{pij}^{(Ind)}$ is the residual for variable$p$, *n* is the total number of respondents, and *N* is the total number of households. We assume that $\eta_{ij}^{(Ind)}$ $\sim N \left( 0, \psi^{(Ind)} \right)$ and $\eta_{j}^{(HH)}$ $\sim N \left( 0, \psi^{(HH)} \right),$ while each of the twelve observed variables residuals, $e_{pij}^{(Ind)}\sim N \left( 0, \theta_{p}^{(Ind)} \right)$, where $\psi^{(Ind)}$ , $\psi^{(HH)}$and $\theta_{p}^{(Ind)}$ are the covariance matrices of the latent individual psychiatric morbidity, latent household psychiatric morbidity, and the residuals of the *p*^th^ observed indicator respectively.

*A1.2 Structural component*

To model the effect the co-residents’ history of mental disorder has on an individual’s psychiatric morbidity we have the following set of structural equations:

$$\eta_{ij}^{\left( Ind \right)}= \eta_{j}^{\left( HH \right)}+\beta_{co}X_{ij}^{\left( co \right)}+\sum_{k=1}^{7} \beta_{k}X_{k} +\xi_{ij}^{\left( Ind \right)}$$

$$\forall i=1,2,..,n, j=1,2,,N , K=1,..,7 (2)$$

Where $X_{\mathrm{ij}}^{(co)}$ is the co-residents’ history of mental disorder for individual *i* in the *j^th^* household, and $X_{k}$ is the *k*^th^ predictor that is hypothesized to affect an individual’s psychiatric morbidity at the individual-level. The error term at the individual-level, $\xi_{ij}^{(Ind)}\sim N \left( 0, \psi^{(Ind)} \right)$.

To explain the variability in psychiatric morbidity between households we regress $\eta_{j}^{(HH)}$ on two predictors measured at the household-level: the household deprivation index ($Z_{1j})$ and having close relationship with other people as aggregated to the household level ($Z_{2j})$.

The household-level equation is formulated as follows:

$\eta_{j}^{(HH)}=\gamma_{0}+\sum_{r=1}^{2} \gamma_{r}Z_{r}+\delta_{j}^{(HH)},$ (3)

Where $\gamma_{0}$ is the overall intercept, $\gamma_{r}$ is the *r*^th^ slope parameter associated with the observed variable $Z_{r}$ and $\delta_{j}^{(HH)}$ is the error term such that $\delta_{j}^{(HH)}\sim N \left( 0{,\psi}^{(HH)} \right)$.

Substituting equation (3) into equation (2), the second set of structural equations can be represented as follows:

$\eta_{ij}^{\left( Ind \right)}= \gamma_{0}+\beta_{co}X_{ij}^{\left( co \right)}+\sum_{k=1}^{7} \beta_{k}X_{k}+\sum_{r=1}^{2} \gamma_{r}Z_{r} +(\xi_{ij}^{\left( Ind \right)}{+ \delta}_{j}^{\left( HH \right)})$(4)

Thus, equation (4) now captures the variance at an individual-level and a household-level $(\xi_{ij}^{\left( Ind \right)}{+ \delta}_{j}^{\left( HH \right)})$. Essentially, equation (4) describes the *i*^th^ person’s unobserved psychiatric morbidity score in terms of their personal characteristics and their household characteristics while the residual (unexplained) variance is partitioned into two parts: (a) the unexplained deviation of an individual’s score from other individuals within the same household ($\xi_{ij}^{\left( Ind \right)})$ and; (b) the unexplained deviation of the individual’s score from other individuals that belong to different households ($\delta_{j}^{\left( HH \right)})$.

From the model, represented by equations (1) and (4), we are interested in estimating the parameters’ matrices of two groups of structural equations. For the measurement component equation (1), we estimate the factor loadings ($\lambda)$, the variance of the latent variables ($\psi$*)*, the measurement error variances and their covariance ($\theta$). For the regression part, equation (4), we are interested in estimating the regression parameters ($\beta)$and$(\gamma)$ .

Thus, the proposed multilevel structural equations model allows us to study the effects the co-residents have on an individual’s psychiatric morbidity. The co-residents’ effect is captured in two ways: first we examine the effect of living with at least one co-resident who has previously experienced a common mental disorder; second, we consider the household level to explain the dependency of psychiatric morbidity for all household members. Thus accounting for the multilevel structure of the data allows us to explain dependency of psychiatric morbidity among individuals within the same household. In other words, we were able to explain the dependency between each individual and his/her co-residents.
